# Wedelolactone disrupts the interaction of EZH2-EED complex and inhibits PRC2-dependent cancer

**DOI:** 10.18632/oncotarget.3790

**Published:** 2015-04-12

**Authors:** Huiming Chen, Shijuan Gao, Jiandong Li, Dong Liu, Chunjie Sheng, Chen Yao, Wei Jiang, Jiaoxiang Wu, Shuai Chen, Wenlin Huang

**Affiliations:** ^1^ CAS Key Laboratory of Pathogenic Microbiology and Immunology, Institute of Microbiology, Chinese Academy of Sciences, Beijing 100101, China; ^2^ School of Life Sciences, Anhui University, Hefei 230039, China; ^3^ Sun Yat-sen University Cancer Center, State Key Laboratory of Oncology in South China, Collaborative Innovation Center for Cancer Medicine, Guangzhou 510060, China; ^4^ The Key Laboratory of Tumor Targeted Medicine in Guangdong Province, Guangzhou Double Bio-product Inc., Guangzhou 510663, China

**Keywords:** epigenetic cancer therapy, surface plasmon resonance, cell cycle, apoptosis, cell migration

## Abstract

Polycomb repressive complex 2 (PRC2), which is responsible for the trimethylation of H3K27 (H3K27me3), plays a part in tumorigenesis, development and/or maintenance of adult tissue specificity. The pivotal role of PRC2 in cancer makes it a therapeutic target for epigenetic cancer therapy. However, natural compounds targeting the enhancer of zeste homolog 2 (EZH2) - embryonic ectoderm development (EED) interaction to disable PRC2 complex are scarcely reported. Here, we reported the screening and identification of natural compounds which could disrupt the EZH2-EED interaction. One of these compounds, wedelolactone, binds to EED with a high affinity (*K*D = 2.82 μM), blocks the EZH2-EED interaction *in vitro*, induces the degradation of PRC2 core components and modulates the expression of detected PRC2 downstream targets and cancer-related genes. Furthermore, some PRC2-dependent cancer cells undergone growth arrest upon treatment with wedelolactone. Thus, wedelolactone and its derivatives which target the EZH2-EED interaction could be candidates for the treatment of PRC2-dependent cancer.

## INTRODUCTION

Cancer is a major public health problem in the world. In the United States, estimated new cancer cases and cancer deaths in 2014 are 1,665,540 and 585,720, respectively [1]. Recurrent somatic mutations in numerous epigenetic regulators in various cancers draw much attention and highlight the fact that we have now entered an era of epigenetic cancer therapies [2, 3]. The epigenomic landscape features different machinery in transcriptionally active versus silent regions [4]. Polycomb group (PcG) proteins, conserved chromatin proteins, are widely deployed in higher eukaryotes to implement gene silencing [5].

Polycomb group (PcG) proteins found in *Drosophila melanogaster* are responsible for homeotic gene (Hox) silencing [6]. Further study discovered a variety of their functions such as participating in mammalian X-chromosome inactivation and imprinting [7, 8], maintenance of pluripotency and self-renewal in embryonic stem cells (ESCs) [9], epigenetic cell cycle control [10], cell fate decisions and developmental controls. PcG proteins mainly function by forming two evolutionarily conserved multimeric protein complexes, Polycomb repressive complexes 1 (PRC1) and Polycomb repressive complexes 2 (PRC2). They are involved in monoubiquitylation of lysine 119 of histone H2A (H2AK119ub) and di- and tri-methylation of lysine 27 of histone H3 (H3K27me3), respectively [11]. PRC2 contains three essential subunits: a catalytic subunit with methyltransferase activity, enhancer of zeste homolog 2 (EZH2) and two noncatalytic subunits, suppressor of zeste 12 (SUZ12) and embryonic ectoderm development (EED). Much attention is paid to their association with sorts of cancers like colon cancer, breast cancer, leukemia, hepatocellular carcinoma and tongue cancer [12-15].

Some groups target PRC2 through inhibiting its core component EZH2. Lots of EZH2 inhibitors are developed including 3-deazaneplanocin A (DZNep), EPZ005687 and GSK126 [16-18]. Others target PRC2 by disrupting the interaction between EED and EZH2. Interaction between EED and EZH2, which is essential to PRC2′s HMTase activity as well as its function [19], serves as an interesting target for drug development. The N-terminal sequence of EZH2 (residues 39–68) mediates its association with EED, among which F42, N45, L56 and V68 are indispensable [20]. An stabilized a-helix of EZH2 (SAH-EZH2) peptide derived from this region (contains residues 40–68) was reported to selectively inhibit H3 Lys27 trimethylation by disrupting the EZH2–EED complex [21]. However, natural compounds targeting the EZH2-EED interaction are scarcely reported.

In this study, we used the Biacore 3000 and competitive co-immunoprecipitation (co-IP) assay to screen for small-molecule inhibitors which could disturb the binding of EZH2 to EED from the natural products library. Two compounds, epigallocatechingallate (EGCG) and wedelolactone, were identified and further studied. Interestingly, EGCG has been reported by Subhasree Roy Choudhury's group with a function to negatively regulate PRC2 [22]. In addition to disrupt PRC2, we found that wedelolactone also induce the degradation of PRC2 core components and modulate the expression of PRC2 targets and cancer-related genes. Moreover, we observed that wedelolactone could inhibit the proliferation and migration, induce cell cycle arrest and apoptosis of PRC2 dependent cancer cells. Our results provide evidences that EZH2-EED interaction is a target for the treatment of PRC2-dependent cancer and wedelolactone is a candidate for modifications in the future.

## RESULTS

### Screen for natural compounds disrupting the EED-EZH2 interaction

**Figure 1:**
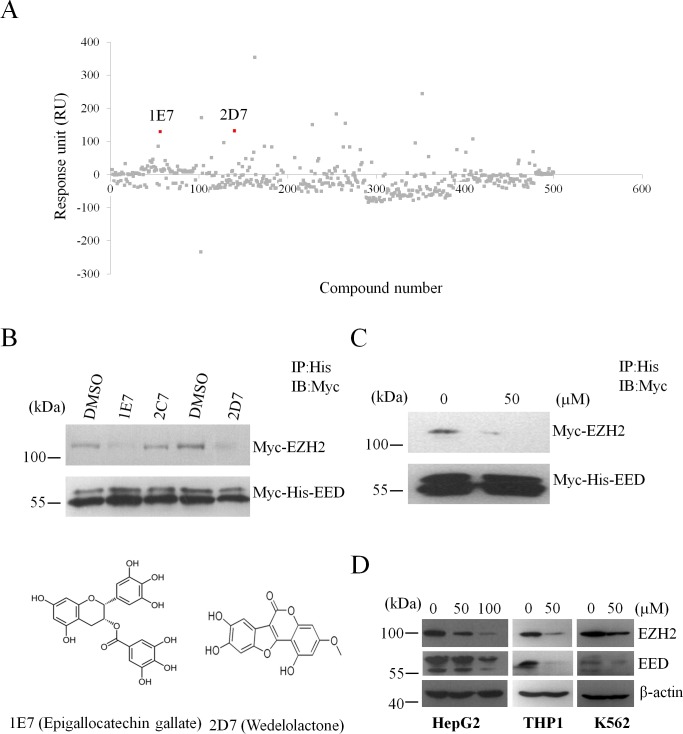
Screen for natural compounds disrupting the EED-EZH2 interaction (**A**) Representative sensorgrams were obtained from injections of natural compounds over the CM5-EED surface. 1E7 and 2D7 refers to epigallocatechingallate and wedelolactone, respectively. (**B**) Competitive co-immunoprecipitation assay was performed with the indicated natural compounds with the concentration of 5μM or DMSO. 2C7 refers to tetrandrine as a negative control. The protein levels of Myc-EZH2 and Myc-His-EED were evaluated by WB with anti-Myc antibody. (**C**) Myc-EZH2 and Myc-His-EED were translated with the reticulocyte lysate system *in vitro* followed by incubation with wedelolactone or DMSO to perform competitive co-IP analysis. (**D**) Wedelolactone depletes PcG proteins. HepG2, THP1 and K562 cells were incubated with the indicated concentrations of wedelolacone for 24 h. The levels of EZH2, EED and H3K27me3 were then analyzed with specific antibodies as indicated.

EED was reported to bind the N-terminal sequence of EZH2 (residues 39-68) [20], so natural compounds which could bind to EED might disrupts the EZH2-EED interaction. Then we used the SPR platform Biacore 3000 to screen for natural compounds that bind to EED. Fresh recombinant EED was covalently immobilized on a CM5 sensor chip as ligand before detection. Natural compounds were diluted in PBS buffer and injected as analyte. The response unit (RU) of each compound was collected and was showed in Figure 1A.

Then, we performed competitive co-immunoprecipitation (co-IP) experiments to identify EED-EZH2 disruptors among natural compounds with RU higher than 50. In these disruptors, we found that 1E7 (EGCG) and 2D7 (wedelolactone) with the concentration of 5 μM could disrupt the interaction between EZH2 and EED significantly (Figure 1B). In order to exclude the potential influence of other proteins in the process, we translated Myc-EZH2 and Myc-His-EED *in vitro* using the reticulocyte lysate system and performed competitive co-IP assays to investigate the effects of 2D7 on the interaction between EZH2 and EED. The results showed that 2D7 blocked the binding of EZH2 to EED efficiently (Figure 1C), suggesting a direct inhibition of 2D7 on the association of these two proteins.

As dismantling the PRC2 complex could result in the decrease of protein stability and further depletion of PcG members [21], we examined whether wedelolactone treatment altered the levels of EZH2 and EED. As shown in Figure 1D, wedelolactone treatment reduced the protein levels of these two core PRC2 components in human hepatocellular carcinoma cell lines HepG2, human monocytic leukemia cells THP1 and human myeloid leukemia cell lines K562.

### SPR detection of EGCG and wedelolactone binding to EED

**Figure 2:**
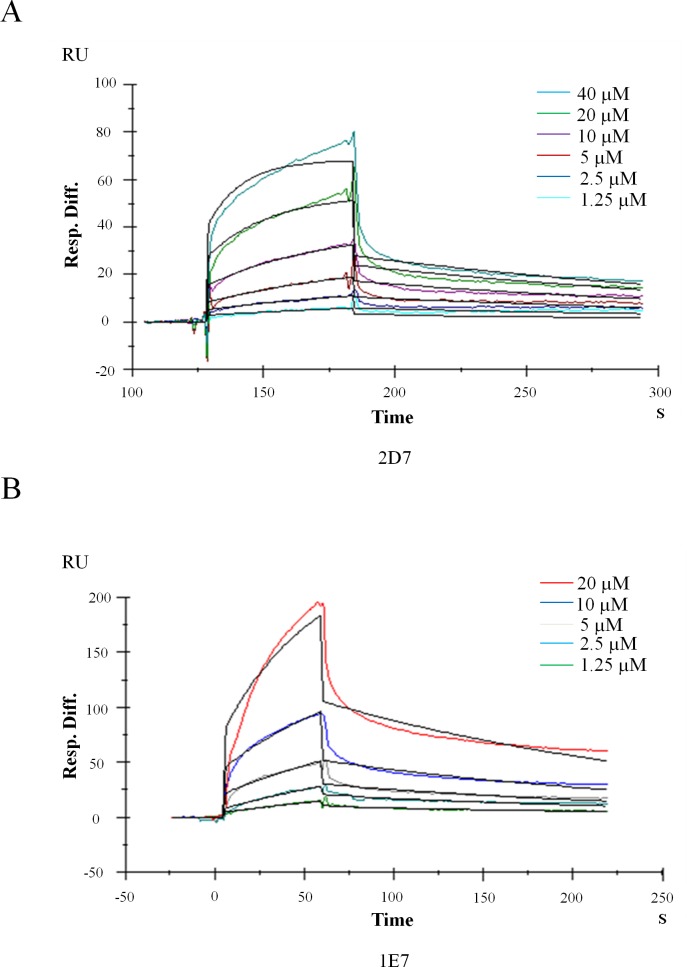
Kinetics analysis of wedelolactone and EGCG binding to EED based on SPR platform Biacore 3000 Representative Sensorgrams were obtained from injections of wedelolactone (**A**) or EGCG (**B**) at indicated concentrations.

Drug candidate is usually expected to bind its target with a high affinity [23]. Here SPR platform Biacore 3000 was used to monitor the direct interaction between wedelolactone/EGCG and EED. Fresh recombinant EED proteins were covalently immobilized on a dextran sensor chip as ligand before detection. Wedelolactone/EGCG was serially diluted in a vehicle of 1% DMSO in PBS buffer and injected as analyte to flow liquid phase. The sensorgrams had shown direct binding between wedelolactone (Figure 2A)/EGCG (Figure 2B) and EED molecule in a dose-dependent manner. Evaluated by BIA evaluation software, the equilibrium dissociation constant (*K_D_*) value of wedelolactone (2D7)/EGCG (1E7) to EED is 2.82 μM and 15.1 μM, respectively (Table 2).

### Wedelolactone modulates PRC2 targets and tumor-related genes expression in PRC2-dependent cancer cells

PRC2 has been reported to participate in silencing a myriad of target genes which are important in tumorigenesis and cancer progression. Several PRC2 target genes harbor tumor suppression function. For example, the DOC-2/DAB2 interactive protein(*DAB2IP*), a growth inhibitor, is involved in the tumor necrosis factor-mediated JNK signaling pathway leading to cell apoptosis [24]; Beta-2 adrenergic receptor(*ADRB2*) activation in xenograft mouse models inhibits prostate cancer tumor growth *in vivo* [25]; Loss of *CDKN2A* contributes to the loss of control over cell cycle, the bypass of critical senescent signals and is associated with progression to malignant disease [26] and *GADD45A* (growth arrest and DNA-damage-inducible, alpha) involves in cell cycle and apoptosis [27].

Dysregulation of EZH2 alters the expression of many cancer related genes [28, 29]. For instance, targeting EZH2 could deplete *HOXA9* and *Meis1* levels in THP1 cells and disrupt the biological synergy between the two genes in inducing myeloid leukemia [30]. Moreover, sensitivity of cancer cells to the EZH2 inhibitors is partly dependent on *PTEN* and *p53* [31, 32].

**Figure 3:**
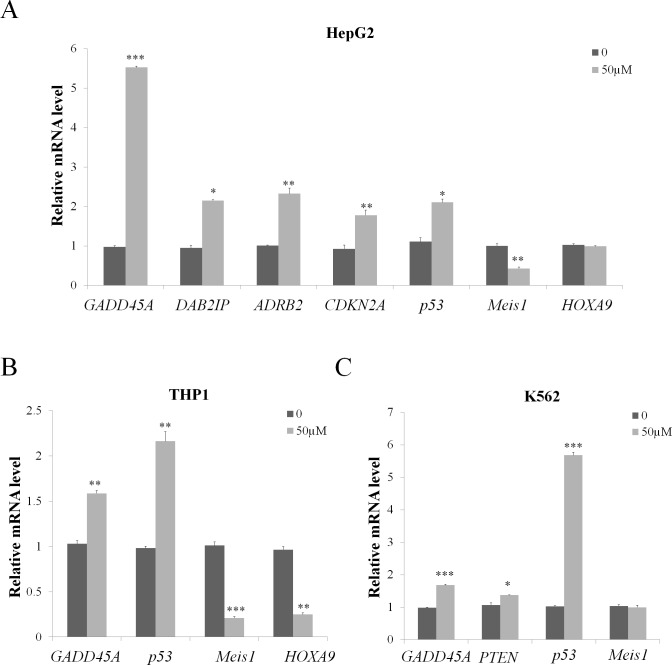
Wedelolactone modulates PRC2 target and tumor-related genes expression in PRC2-dependent cancer cells HepG2 cells (**A**), THP1 cells (**B**) and K562 cells (**C**) were treated with 50 μM wedelolactone for 24 h. Total RNA was isolated and qRT-PCR was performed with specific primers for the indicated target genes. Quantification results were shown as folds of control and expressed as the mean ± SD (*n* = 3). *P < 0.05, **P < 0.01, ***P < 0.001.

To explore the regulation of wedelolactone treatment on the expression of these PRC2 target genes and cancer related genes, HepG2, THP1 and K562 cells were treated with 50 μM wedelolactone for 24 h. Total RNA were extracted and the mRNA levels of the above genes were analyzed by quantitative real-time PCR. As shown in Figure 3A, wedelolactone treatment significantly induces the expression of *GADD45A*, *DAB2IP*, *ADRB2*, *CDKN2A* and *p53* while represses *Meis1* expression in HepG2 cells. As shown in Figure 3B, wedelolactone significantly repressed *HOXA9* and *Meis1* expression while enhances the expression of *GADD45A* and *p53* in THP1 cells. At the meantime, the expression of *GADD45A*, *PTEN* and *p53* were activated after treatment with wedelolactone in leukemia cell lines K562 (Figure 3C). Together, our results indicated that PRC2 targets and tumor-related genes which were involved in apoptosis and cell cycle arrest were modulated by wedelolactone.

**Table 1:** Oligonucleotide sequences used in this study

Primer name	Primer sequence
GAPDH Fl	GAAGGTGAAGGTCGGAGTC
GAPDH RI	GAAGATGGTGATGGGATTC
DAB2IP Fl	ACATCCAGATGAAGGGCATC
DAB2IP RI	GCGTGGTCCTTCTTCTTCAGTTC
ADRB2 F I	AGCCAGTGCGCTCACCTGCCAGACT
ADRB2 RI	GCTCGAACTTGGCAATGGCTGTGA
CDKN2A F I	GCTGCCCAACGCACCGAATA
CDKN2A RI	ACCACCAGCGTGTCCAGGAA
PTEN F I	AATCCTCAGTTTGTGGTCT
PTEN RI	GGTAACGGCTGAGGGAACT
Meisl Fl	CCCTGGAATGCCAATGTCA
Meisl RI	GAGCGTGAATGTCCATGACTTG
P53 Fl	CCAGCAGCTCCTACACCGGC
P53 RI	GAAACCGTAGCTGCCCTG
GADD45A Fl	CGCCTGTGAGTGAGTGC
GADD45A RI	CTTATCCATCCTTTCGGTCTT
HOXA9 Fl	GCTTGTGGTTCTCCTCCAGT
HOXA9 RI	CCAGGGTCTGGTGTTTTGTA

**Table 2:** Kinetic parameters of the binding of 2D7 and 1E7 to EED

	*R_max_*(RU)	*k_on_*(M^−1^s^−1^)	*k_off_*(s^−1^)	*K_D_*(M)	*ϰ^2^*
2D7	30.2	1.82×10^3^	5.13×10^−3^	2.82×10^−6^	2.55
1E7	414	302	4.57×10^−3^	1.51×10^−5^	20.7

*R_max_*, maximum analyte binding capacity; *k_on_*, association rate constant; *k_off_*, dissociation rate constant; *K_D_*, equilibrium dissociation constant. *K_D_* =*k_off_/k_on_*; ϰ^2^, statistical value in Biacore. All parameters were measured according to Langmuir fitting model, using Biacore Evaluation Software (version 4.01).

### Wedelolactone inhibits PRC2-dependent cancer cells

Since human leukemia K562 cells, THP1 monocytes and hepatocellular carcinoma HepG2 cells are partially PRC2-dependent [21, 33, 34], we studied the anti-cancer effect of wedelolactone on these cells.

**Figure 4:**
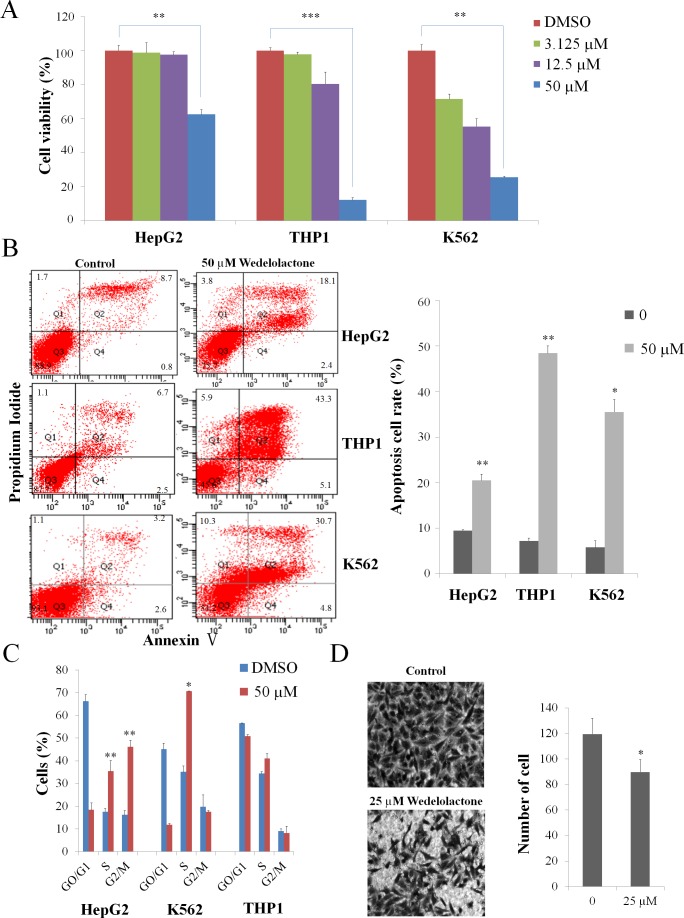
Wedelolactone inhibits PRC2-dependent cancer cells K562 cells, HepG2 cells and THP1 cells were treated for 48 h with the indicated concentrations of wedelolactone. The cells were then harvested. The viability was assayed by MTT assay (**A**) and the apoptosis was assayed by AnnexinV-FITC/PI double staining assay (**B**). (**C**) Cells were treated with 50 μM wedelolactone for 24 h, and the cell cycle distribution was subsequently determined via flow cytometric analysis. (**D**) wedelolactone regulates migration of HepG2 cells. Wedelolactone decreased the number of migration cells compared with control cells (original magnification ×200). Quantification results were shown as folds of control and expressed as the mean ± SD (*n* = 3). *P < 0.05, **P < 0.01, ***P < 0.001.

We first examined the effects of wedelolactone on cell proliferation. As shown in Figure 4A, 50 μM of wedelolactone treatment repressed the proliferation of HepG2, THP1 and K562 cells. Since many drugs have been shown to inhibit cancer cells through induction of apoptosis, we then detected the apoptotic ratio in cells with or without wedelolactone treatment by Annexin V-FITC/PI double staining assay. As shown in Figure 4B, the presence of wedelolactone significantly increased apoptosis in HepG2, THP1 and K562 cells.

It is well-known that most anticancer agents, such as vinblastine and paclitaxel, have been documented to arrest cell cycle [35]. So we examined the effect of wedelolatctone on cell cycle distribution of HepG2, THP1 and K562 cells. Compared with control, there was an accumulation of cell population in S and G2/M phase after wedelolactone exposure in HepG2 cells and the proportion of K562 cells in S phase were increased after wedelolactone exposure (Figure 4C). However, it exhibited no significant effect on THP1 cells.

Recently, several studies about EZH2 regulating cell invasion in various types of cancer showed that one of the major EZH2 PRC2-dependent function is promoting cell invasion [36-38]. Additionally, the migration of K562 and THP1 cells are scarcely reported. So we interrogated the effect of wedelolactone on the migration of HepG2 cells.

To determine whether wedelolactone inhibits cell migration in HepG2 cells, we performed Transwell migration assays. As shown in Figure 4D, the presence of wedelolactone significantly suppressed cell migration in HepG2 cells. Together, our data suggested that wedelolactone inhibited proliferation, induced apoptosis and cell cycle arrest and suppressed cell migration of PRC2-depedent cancer cells.

## DISCUSSION

Wedelolactone is an essential active compound of Eclipta prostrate. It has been reported to possess various biological functions, including the inhibition of IKK kinase, K+-ATPase activity, hepatitis virus C RNA-polymerase, phospholipase A2, 5-lipoxygenase and DNA topoisomerase IIα [39]. Also, it exhibits anti-cancer function in some cancers, such as prostate cancer, breast cancer and so on [39]. The growth inhibition effects of wedelolactone on tumor cells were believed to be accomplished through its inhibition of IKK, the androgen receptor [40], or topoisomerase II. But its function on the EED-EZH2 interaction and the PRC2 activity is unknown.

In the present study, we present a new method to identify inhibitors targeting PRC2. Firstly, Biacore 3000 was used to screen for natural compouds which could bind to EED. Then the competitive co-IP experiment was performed to further identify PRC2 disruptors. By this way, we identified that EGCG and wedelolactone could bind to EED and target the EZH2-EED interaction.

Tumor suppressors refer to a large group of molecules that can prevent cancer through controlling cell division, promoting apoptosis, helping DNA damage repair and suppressing metastasis [41]. So reactivating tumor suppressors which are silenced by PRC2 will contribute to the inhibition of carcinoma cells proliferation. Indeed, wedelolactone could activate PRC2 downstream tumor suppression genes such as *DAB2IP*, *ADRB2*, *CDKN2A* and *GADD45A* (Figure 3A), thus it serves as a mechanism for its inhibition on PRC2-dependent cancer cells. In fact, not all the target genes can be influenced by wedelolactone (data not shown).

In conclusion, we identified that wedelolactone could bind to EED and target PRC2, thereby modulate its targets and cancer-related genes. As a consequence, wedelolactone exhibits anti-cancer effects by inducing proliferation and migration inhibition, apoptosis and cell cycle arrest of PRC2-dependent cancer cells. So it could serve as a candidate for the treatment of PRC2-dependent cancer. Also, our work verified the possibility to the development of anti-cancer agents by disrupting the association of core PRC2 components EZH2 and EED.

## MATERIALS AND METHODS

### Plasmids

The EZH2 gene was amplified by PCR and subcloned into pcDNA3.0-Myc (Invitrogen). The EED gene was amplified by PCR and subcloned into pcDNA4.0-Myc/His (Invitrogen) and pGEX-4T-1 plasmid (GE Healthcare) respectively.

### Abs and reagents

The primary antibodies used in this study were as follows: anti-Myc (sc-40), anti-EED (sc-28701) and anti-β-actin (sc-47778) were purchased from Santa Cruz Biotechnology. Anti-EZH2 (#3147S) was from Cell Signaling Technology, anti-His (#TA-02) was from ZSGB-BIO and anti-trimethyl histone H3 (Lys27) antibody (ABE44-S) was from Millipore. Wedelolactone was purchased from National Institutes for Food and Drug Control (NIFDC, China). Dimethylsulfoxide (DMSO) was obtained from Sigma-aldrich (USA). Protein G beads and GST beads were purchased from Santa Cruz Biotechnology and GE Healthcare, respectively.

### Cell culture

HepG2, K562 and 293T cells were cultured in DMEM (Invitrogen) supplemented with 10% FBS (Hyclone), 100 U/ml penicillin and 100 μg/ml streptomycin. THP1 cells were cultured in RPMI-1640 supplemented with 10% FBS, 100 U/ml penicillin, 100 μg/ml streptomycin and 2 mM L-Glutamine. All cell lines were maintained at 37°C under a 5% CO_2_ atmosphere. Transfection of cells was performed by using Entranster-H (Engreen, China) according to the manufacturer's instructions.

### Binding detection based on SPR platform

The interaction between compound and protein was detected by surface plasmon resonance platform Biacore 3000 (GE Healthcare). Fresh EED protein was diluted to 100 μg/ml in 10 mM acetate buffer (pH 5.0), and then immobilized as ligand in the NHS/EDC pre-activated CM5 sensor chip, following blocking by ethanolamine. Final amount of protein immobilization reached 10000 RU. The compound stock was diluted in a vehicle of 1% DMSO (v/v) in phosphate buffered saline (PBS). The dilutions were injected as analyte flow liquid phase with PBS containing 1% DMSO (v/v) as running buffer at a constant flow rate of 30 μl/min. Ninety seconds' association time was set, followed by 180 s dissociation time. All buffers in the experiment were subjected to be filtered by 0.22 μm filters and degassed by ultrasonic. The data were collected by Biacore Control Software (version 4.1.1). Kinetics and affinity parameters were evaluated in Langmuir model (1:1) by using BIA evaluation software (version 4.1).

### Competitive co-immunoprecipitation assay

Cell lysates from 293T cells transfected with Myc-EZH2 and Myc-His-EED were incubated with anti-His antibody, protein G beads and natural compound with corresponding concentration or DMSO overnight at 4°C. The beads were then washed three times and boiled to be used for WB.

### *In vitro* translation assays

Myc-EZH2 and Myc-His-EED were translated *in vitro* with TNT T7 coupled reticulocyte lysate system (Promega, #L4611) according to the manufacturer's instructions. The *in vitro* translated products were used to perform competitive co-immunoprecipitation assay.

### Western blotting

Cells were lysed and prepared with 1 × SDS Reducing sample buffer (CST, #7722) according to the manufacturer's instructions. Appropriate volume of sample was loaded onto the SDS-polyacrylamide gels and transferred to a PVDF membrane. After blocking, the membrane was incubated with the primary antibody overnight at 4°C followed by incubation with a horseradish peroxidase-conjugated secondary antibody for 2 h at room temperature. Bands were detected using enhanced chemiluminescence (Applygen, China).

### RNA extraction and quantitative reverse transcriptase polymerase chain reaction (qRT-PCR)

Total RNA was isolated from the cells using TRNzol (TIANGEN, China). cDNA was synthesized using the RevertAid First Strand cDNA Synthesis kit (Thermo Scientific, #K1622). Quantitative real-time PCR (qRT-PCR) was conducted using SYBR premix Ex Taq II (Takara, China). Thermal cycling was performed using an ABI 7300 real-time PCR machine (Applied Biosystems) as follows: 95°C for 30 s followed by 40 cycles of amplification for 5 s at 95°C, 31 s at 60°C. The primer sequences used for PCR are listed in Table 1.

### Measurement of cell viability

Cell viability was determined by MTT Cell Proliferation and Cytotoxicity Detection Kit (KGA312, KeyGEN BioTECH, China). A total of 5×10^3^ cells were seeded in 96 well plates. At 48 h post-treatment with different concentrations of wedelolactone, 1×MTT was added to the wells and incubated for additional 4 h at 37 °C. The optical density of the dissolved material was measured at 490 nm.

### Cell cycle analysis

The effect of wedelolactone on cell cycle distribution was determined by flow cytometric analysis. The cells were treated with 50 μM wedelolactone for 24 h. Appropriate controls were also set up. After treatment, 1×10^5^ floating and adherent cells were collected, washed with PBS and fixed with 70% ethanol. Staining for DNA content was performed using Cell Cycle Detection Kit (KGA512, KeyGEN BioTECH, China). Populations in G0/G1, S and G2/M phases were measured by BD FACSCalibur Flow Cytometry System with CellQuest Pro software (BD Bioscience). Data were analyzed using the ModFit 3.0 Software.

### Detection of apoptotic cells by flow cytometry

Cells were plated in six-well plates at a density of 1×10^5^ cells/ml and incubated overnight. Wedelolactone or DMSO was then added into each well and incubated for 48 h. Cells were collected and washed with PBS, followed by resuspension in 300 μl binding buffer at a concentration of 5×10^5^ cells/ml. Mixed with Annexin V-FITC and propidium iodide (PI) according to the manufacturer's instructions. The mixed solution was incubated in the dark at room temperature for 15 min. Cell apoptosis analysis was performed using the BD FACSAriaII Flow Cytometry System (BD, USA) within 1 h. Data were analyzed using the FACSDiva Version 6.1 Software.

### Cell migration assay

HepG2 cells were treated with 25 μm wedelolactone or DMSO for 12h then the cells were trypsinized and replated onto the upper chamber of a Transwell filter with 8 μm pores (Costar) at 2×10^5^ cells/well in serum-free medium. Medium supplemented with 10% FBS was placed in the bottom well, and the cells were then incubated for 24 h at 37°C in a humidified 5% CO_2_ atmosphere. After the incubation, the chambers were removed, and migration cells on the bottom side of the membrane were fixed with methanol for 15 min and stained with gentian violet for 10 min. Each experiment was performed in triplicate, and the number of cells in five random fields on the underside of the filter was counted and averaged. The results were expressed as the migrated cell number.

### Statistical analysis

The data are presented as mean ± stand deviation (S.D.). Parametrical data were compared using Student's *t* test. One-way ANOVA analysis was used to determine the difference between independent groups. The differences between the variants were considered to be statistically significant if *P* < 0.05.
